# Advanced methods for insect nets: red-colored nets contribute to sustainable agriculture

**DOI:** 10.1038/s41598-024-52108-1

**Published:** 2024-02-14

**Authors:** Susumu Tokumaru, Yoshiaki Tokushima, Shun Ito, Terumi Yamaguchi, Masami Shimoda

**Affiliations:** 1 Kyoto Prefectural Agriculture, Forestry and Fisheries Technology Center, Kameoka, Kyoto 621-0806 Japan; 2 Matsudo Chiba, 270-2204 Japan; 3 Graduate School of Agricultural and Life Sciences, The University of Tokyo, Bunkyo-ku, Tokyo, 113-8657 Japan

**Keywords:** Environmental biotechnology, Plant biotechnology

## Abstract

Development of advanced pest control methods that do not rely on insecticides is an important issue for sustainable agriculture. Particularly with regards to micro pests that are not only highly resistant to various insecticides but also because we are running out of options for which insecticide to use against them, resulting in enormous economic damage worldwide. Here we report that the effectiveness of the conventional insect net can be greatly advanced by changing their color to red that helps significantly reduce pesticide use. We demonstrate the red effect using Onion thrips, *Thrips tabaci* a main vector of Iris Yellow Spot Virus (IYSV) and Tomato Spotted Wilt Virus (TSWV) that cause serious damage to various vegetables. New red nets succeeded in suppressing the invasion rates and damages (white spots on the leaves) in a Welsh onion greenhouse with minimum use of pesticides. We discuss how red nets are compatible with labor-saving, sustainable agriculture and the future potential of “optical pest control” based on insect color vision and its behavioral response.

## Introduction

In agricultural production, pest control is as much a factor harming Earth’s environment as chemical fertilizers and fossil fuels. Therefore, new technologies and ideas are needed for pest control to support sustainable food production. Since the time pest control relying on powerful synthetic insecticides started in the 1960s, a control method that combines different environmentally friendly techniques called Integrated Pest Management, IPM, has been explored^1,2^. For example, an IPM method combining natural predators and attractants including pheromones, has been used to suppress pest densities below economically acceptable levels. Another example is insect nets, which have been traditionally used in agricultural production just like mosquito nets are popularly used for protection from *Anopheles* mosquitoes in bedrooms^3,4^. In agriculture, insect nets generally use a fine mesh to “physically” prevent pests from entering cultivation facilities^5–8^. The red colored insect net presented in this paper is a novel pest control method that is based on color recognition by the insect compound eye. Even though the red insect net has a mesh size that is larger than the pest body, it is more effective than conventional insect nets indicating that it works on a different principle.

Until now, the net color was not considered in product development because the general notion was that it is sufficient to make the mesh size small enough that pests cannot physically pass through. Therefore, conventional insect nets have been produced using mainly colors such as white, black, and blue out of convenience. However, if the mesh size is too small, there are trade-offs such as reduced exposure of crops to sunlight that results in lower yields, as well as reduced on that can lead to local environment deterioration due to higher temperatures and fungal infection^9,10^.

In 2015, an interesting phenomenon was discovered in insect behavior. It was observed that thrips density decreases when crops are irradiated with red light (wavelength 650–750 nm), which is invisible to these pests^11^. At that time, this phenomenon was not expected to lead to a completely new pest control technology. However, as a result of improvements over the years^12–15^, we have succeeded in developing a new concept with insect nets that effectively prevents pest invasion and crop damage in cultivation facilities. Since the mesh size can be larger than the size of pests, it is also possible to increase light transmittance and reduce incidence of mold diseases such as powdery mildew.

**Figure 1:**
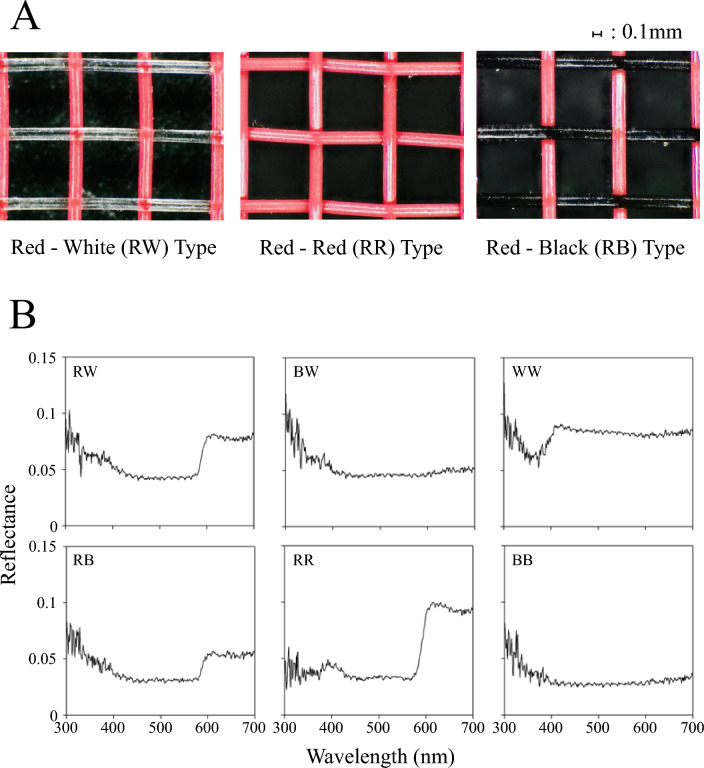
(**A**) Red–white, red-red, and red–black nets used in the experiment. Red–white netwoven with red polyethylene thread in warp and transparent polyethylene thread in weft; Red-red net—woven with red polyethylene thread in both the warp and weft; and Red–black net: woven with red polyethylene thread in warp and black polyethylene thread in weft. (**B**) The reflectance spectra of nets used in the experiments.

Onion thrips *Thrips tabaci* Lindeman (Thysanoptera: Thripidae) are known to be one of the most important agricultural pests worldwide. It causes serious damage to Welsh onion, onion, persimmon, and citrus^16^ in two ways. First, *T. tabaci* acts as a vector of Iris Yellow Spot Virus (IYSV) and Tomato Spotted Wilt Virus (TSWV)^17,18^. Second, it has developed strong resistance to the insecticides including pyrethroids^19–21^. Especially in the genotype of arrhenotokous, on which only a few insecticides remain to be effective in Japan^22–26^. In this study, we prepared three new types of red nets and examined the behavioral response of *T. tabaci* against them in comparison with commercially available white/black nets. Next, we used them for the control of Welsh onion pests under field conditions. We present results showing that the red insect net can contribute significantly to labor-saving and sustainable agricultural production and discuss the potential of “optical pest control” based on the nature of insect color vision (Fig. 1).

## Results

### Behavioral responses of *Thrips tabaci* against the red-colored nets on Welsh onion

**Figure 2:**
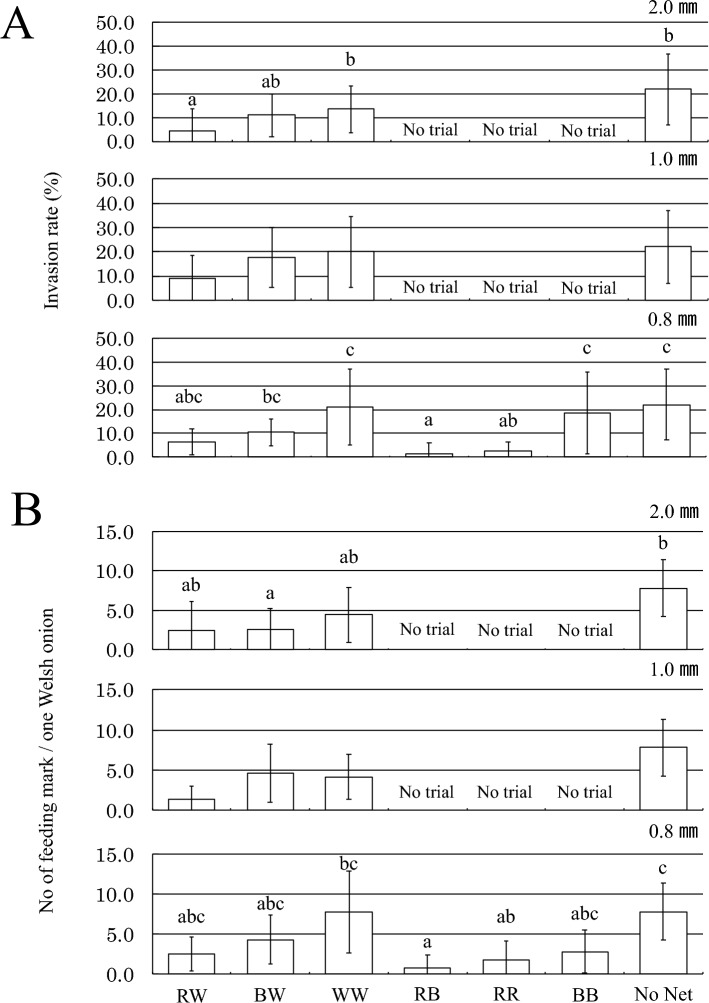
(**A**) Comparison of the pest control effect of the six types of insect nets used against *Thrips tabaci* female adults. There is a significant difference between samples designated with different letters (Tukey–Kramer multiple comparison test using arcsine transformed values, *p* < 0.05). (**B**) Comparison of the effectiveness of the six types of insect nets in controlling the damage caused by *Thrips tabaci* female adults. There is a significant difference between samples designated with different letters (Steel–Dwass multiple comparison test, *p* < 0.05).

Among the 2.0 mm mesh nets, the red-white nets reduced thrips invasion into the net by one-third compared with the white nets, which was a significant difference (Fig. 2A). In the case of the 1.0 mm mesh nets, the red-white nets reduced thrips invasion to about one-half of the white nets, but this difference was not significant (Fig. 2A). In the 0.8 mm mesh nets, the red–black nets and the red-red nets significantly reduced thrips invasion to about 1/14 and 1/8, respectively, compared with the white nets (Fig. 2A). The difference between the two red-containing nets and the white nets was statistically significant (Fig. 2A). In addition, among the 2.0- and 1.0-mm mesh nets, the red-white nets produced fewer feeding marks than the white nets, but the difference was not significant (Fig. 2B). On the other hand, in the 0.8 mm mesh nets, the number of feeding marks was reduced to about one-ninth to one-fourth in the red–black and red-red nets compared with the white nets, and difference between red–black and white nets was statistically significant (Fig. 2B). In the analysis of the invasion rate and color of the yarn, the effects of the red yarn and the black yarn were significantly different from zero, and the absolute value of the coefficient was higher in the red yarn than in the black yarn (Table S2).

**Figure 3:**
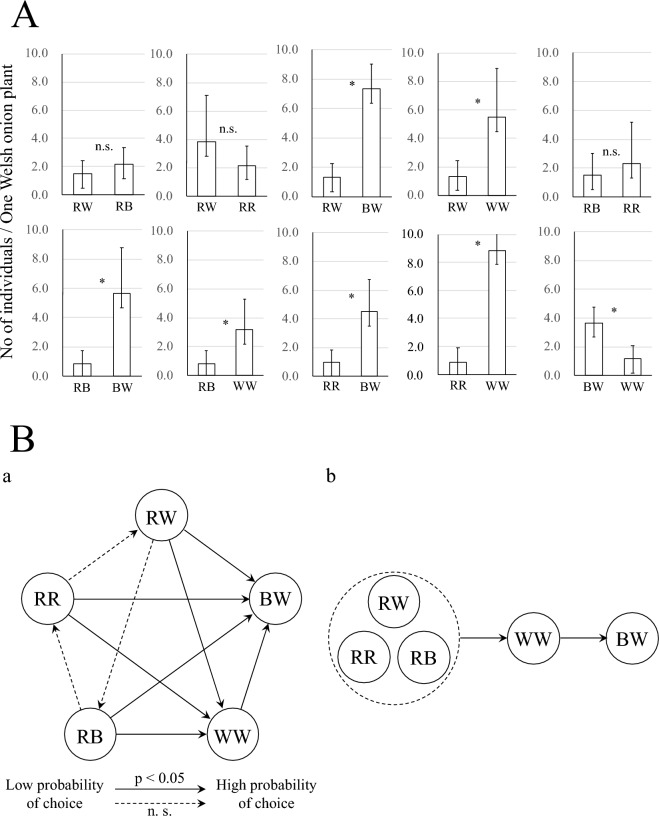
(**A**) Results of the thrips invasion experiments in which two types of insect nets were placed together. Asterisks indicate that the invasion rate is significantly different from 0.5 (Wald test, *p* < 0.05, Refer to Table S3); n.s.,  Not significant. (**B**) Summary of results of the two-net-choice experiments (Fig. 3A, Table S3). (a) Choice probabilities are represented with arrows that point from the net with a smaller probability of choice to the net with a larger probability of choice. Statistically significant probabilities of choice rates are represented as solid lines and not significant ones (n.s.) as dashed ones. (b) A simple visual representation of the relationships “greater than” and “less than”. Nets grouped by the dashed circle represent choice probabilities that are not significantly different from one another, or that their relationships “greater than” and “less than” are transitory. In addition, nets were sorted to avoid inconsistencies in the “greater than” and “less than.” Incidentally, this representation can be reworded in computer science terms^27^ as follows: the graph (i.e., pair of nodes and directed edges) shown in Fig. 3B(a) is decomposed into its strongly connected components and topologically sorted as shown in Fig. 3B(b).

The results of the thrips invasion experiments when two types of insect nets were placed together are shown in Fig. 3A and their statistical analysis is shown in Table S3. As is clearly apparent, in all combinations of red-colored insect nets and black-white or white nets, the number of individuals entering the red-colored net was significantly lower. No significant differences were observed in any of the combinations of red-colored nets. In the combination of black-white and white nets, the number of individuals in the white net was significantly lower. Those results are summarized graphically in Fig. 3Ba and in a simplified form in Fig. 3Bb. From the charts in Fig. S2, the relationships “greater than” and “less than” in the choice probability of two-net-choice experiments can be summarized as RR, RW, RB < WW < BW.

### Control of *Thrips tabaci* by covering Welsh onion fields with red-colored nets

**Figure 4:**
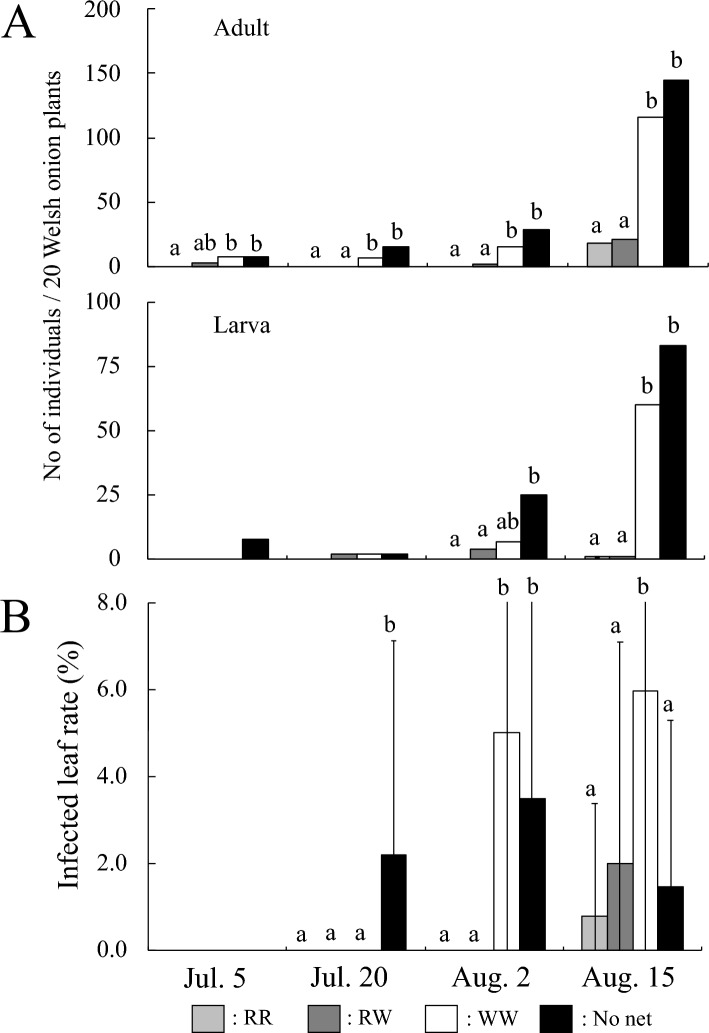
(**A**) The effect of red insect nets on the density of *Thrips tabaci* per 20 Welsh onion plants in Trial 1. Columns denoted by different letters are significantly different at the 5% level by Steel–Dwass multiple comparison test performed at each survey date. (**B**) The effect of red insect nets on IYSV infected leaf rate in Trial 1. Columns denoted by different letters are significantly different at the 5% level by Tukey–Kramer multiple comparison test using arcsine transformed values from each survey date.

In Trial 1, *T. tabaci* outbreaks in white nets and in the no net plots started on 5 July and their populations increased until 15 August (Fig. 4A). In comparison, on 15 August, the adult and larval populations in the red-red net and red-white net plots were significantly reduced to about one-sixth and one-sixtieth, respectively, of those in the white net plots (Fig. 4A). There was no significant difference in the adult and larval populations in red-red net and red-white net plots throughout the study period (Fig. 4A).

With respect to leaf damage rate, there were significant differences between the red-red net and the red-white nets plots compared to the white net and the no net plots (Fig. S3). As can be seen in the figure, on 2 August the damage rate in the red-red net and the red-white net plots was reduced to about 1/21 and 1/9, respectively of that in the white net plots, respectively. Furthermore, in the red-red net and red-white net plots, chlorotic spots was not observed on Welsh onion leaves until 15 August, while leaves with IYSV disease were observed from 20 July in the untreated plot and from 2 August in the white net plots (Fig. 4B). The leaf infection rate in the red-red net and red-white net plots was significantly lower than that in the white net plots on 15 August, about one-seventh and one-third lower, respectively (Fig. 4B).

**Figure 5:**
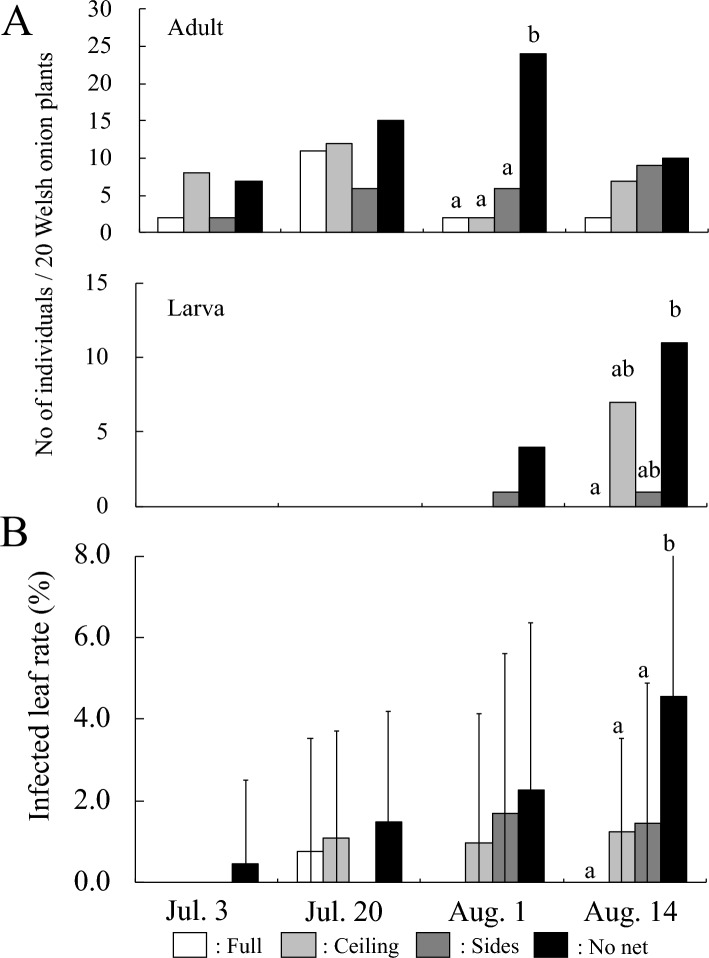
(**A**) The effect of red insect nets on the density of *Thrips tabaci* per 20 Welsh onion plants in Trial 2. Columns denoted by different letters are significantly different at the 5% level by Steel–Dwass multiple comparison test performed at each survey date. (**B**) The effect of red insect nets on IYSV infected leaf rate in Trial 2. Columns denoted by different letters are significantly different at the 5% level by Tukey–Kramer multiple comparison test using arcsine transformed values from each survey date.

In Trial 2, *T. tabaci* adult outbreak began on 3 July and the adult populations in full-, ceiling-, and sides-covering net plots on 1 August was significantly lower than in the no net plots (Fig. 5A). Larvae were not observed in full-covering net plots during the survey period (Fig. 5A). While there was no significant difference in the number of larvae among ceiling-covering, sides-covering, and no net plots, the larval populations in sides-covering plots were reduced to about one-tenth of that in the no net plot (Fig. 5A).

The leaf damage rate in the full-covering net plots was significantly different from that in the no net plots throughout the trial period (Fig. S4). On the other hand, leaf damage rates in the ceiling- and sides-covering net plots remained higher than in the full-covering net plots, and significantly lower than in the no net plots on 14 August (Fig. S4).

IYSV infected leaves were not observed in the full-covering net plots (Fig. 5B). In ceiling- and sides-net plots, the leaf infection rate remained lower than in the no net plots, and on 14 August, it was significantly reduced to about one quarter and one third of the rate in the no net plots (Fig. 5B).

## Discussion

Insect nets are well-known for their use as one of the physical control techniques against thrips, whiteflies, and leaf miners in the cultivation of vegetables. The effect of insect net mesh size on pest invasion rate has been investigated, but the effect of net color has not been studied at all. Our results of the two nets choice experiments (Fig. 3A) suggest that thrips visually discriminate nets containing red fibers (Fig. 3B). The compound eye of *T. tabaci* is thought to be comprised of three spectral types of photoreceptors: UV-sensitive, blue-sensitive, and green-sensitive^28^. Because the light environment in the laboratory does not include UV light, the photoreceptor sensitive to UV light is unlikely to be involved in the mechanism underlying the pest control effect of the red nets; blue and green-sensitive photoreceptors are likely to be involved. At wavelengths longer than 600 nm, spectral reflectance is higher in the red nets (red-red, RR; red-white, RW; and red–black, RB) than in the other nets (Fig. 1B). Therefore, the pest controlling effect could be due to the long-wavelength component of the reflected light from the net stimulating the green photoreceptor cells in thrips. The pest control effect of other reported red light and materials on thrips^11,15,29,30^ could also be explained by the blue- and green-sensitive photoreceptors as well, but further analyses are needed. Ohya et al.^15^ have reported on the effectiveness of red nets in controlling insects. However, they found that the infestation-suppression effect of insect nets was enhanced by the color combination of the net threads. In this study, we selected the best combination of thread colors that suppress pest infestation and statistically proved the principle that red nets inhibit pest infestation.

*T. tabaci* has several reproductive types, and arrhenotokous and thelytokous types have been identified in Japan^19,31^. Arrhenotokous types have developed resistance to synthetic pyrethroids^8,23,24^ and the insecticide susceptibility of arrhenotokous is lower than that of thelytokous^26^. Thus, insecticide-independent pest control techniques are urgently needed.

Red insecticidal nets can be a new physical control technology. Since *T. tabaci* migrate onto the vegetable fields from the surrounding weeds, such as *Stellaria neglecta*, *Stellaria media var. procera*, *Lamium amplexicaule* L., etc.^16,32^, putting up red-colored nets at the openings of greenhouses and around the fields can also significantly reduce *T. tabaci* invasion. In this study, red-colored nets effectively kept the population of *T. tabaci* on leaves low, consistent with report in Ohya et al.^15^. In addition, we found a lower rate of leaf damage (Figs. S3 and S4) and IYSV infection (Figs. 4B and 5B) due to suppression of thrips populations. The reduction in the infected leaf rate in the no net plots was likely due to the high rate of leaf damage caused by *T. tabaci,* and the fact that chlorotic spots are difficult to see in this situation and therefore missed.

In Trial 2, insecticide was sprayed twice, and as a result the full-covering net plots produced Welsh onion with high commercial value by keeping the density of *T. tabaci* infestation lower than the other test plots (Table S4). By comparison, in the no net plots the density of *T. tabaci* was the highest among the four test plots, suggesting that at least twice as many insecticide applications are required to produce Welsh onion with high commercial value. The ceiling- and sides-covering plots also required at least one additional spraying compared to the full-net plots. From these results we conclude that the use of red-colored nets can reduce the number of insecticide applications in the field by 25–50%, making them a significant advancement in labor-saving and sustainable pest control technology.

Welsh onion is infested with not only *T. tabaci*, but also *Frankliniella occidentalis* (Pergande)^33^ and *Liriomyza chinensis* (Kato)^34,35^. Tomato, cucumber, and other vegetables grown in greenhouse in Japan are infested by *F. occidentalis*^33^, *Frankliniella intonsa* (Trybom)^33^, *Bemisia tabaci* (Gennadius)^36^, *Trialeurodes vaporariorum* (Westwood)^37^, *Liriomyza sativae* Blanchard^38^, and *Liriomyza trifolii* (Burgess)^39^. Of these pests, *F. occidentalis*^40–42^, *B. tabaci*^43^, *L. sativae*^44^, and *L. trifolii*^45^ have developed resistance to insecticides, and therefore non-insecticide techniques are required to control them. Our results reported here suggest that it is necessary to investigate the effect of red-colored nets on these pests in detail.

The effects of light on crops have been reported to increase anthocyanin in apples^46^ and promote fruit coloring in strawberries^47^. Shahak et al.^48^ stated that the fruit weight and quality of peaches increased when covered with red-colored nets. The effect of red-colored nets on the growth and yield of Welsh onion is still unknown and needs to be investigated. In our laboratory experiments, red-colored nets with large meshes also suppressed the *T. tabaci* invasion (Fig. 2A) and showed that the red thread is significantly more effective in suppressing the pest infestation than white and black nets (Table S3). It has been pointed out that insect nets deteriorate the cultivation and working conditions by increasing the temperature inside the greenhouse due to reduced breathability^9,10^. Our results indicate that this problem could be solved using red-colored nets with a larger mesh. Further field trials using red-colored nets with larger meshes should be carried out.

## Materials and methods

### Laboratory tests

#### Insects

A laboratory population of *T. tabaci* was established from individuals that were collected from the fields of Welsh onions located in the Kameoka City, Kyoto Prefecture, on 4 August 2015. The laboratory culture was maintained on broad beans, *Vicia faba*, at 25 °C under a 15L–9D photoperiod regime, as described by Sogo et al.^49^ and Aizawa et al.^21^.

#### Insect nets

In the experiments, the following six types of colored insect nets were tested: a plain-woven insect net with red polyethylene yarn in the warp and transparent yarn in the weft with (0.8, 1.0, and 2.0 mm mesh sizes; see red-white net in Fig. 1A), net with red yarn in the warp and black yarn in the weft (only 0.8 mm mesh size; see red–black net in Fig. 1A), net with red yarn in the warp and red yarn in the weft (only 0.8 mm mesh size; see red-red net in Fig. 1A), net with black yarn in the warp and transparent yarn in the weft (0.8, 1.0, and 2.0 mm mesh sizes; black–white net), net with black yarn in both warp and weft (only 0.8 mm mesh size; black and black net), and net with transparent yarn in both warp and weft (0.8, 1.0, and 2.0 mm mesh sizes; white net). Figure 1B shows the spectral reflectance of each type of net measured with a spectrometer via optical fiber connected with an integrating sphere (HS1000S, Asahi Spectra Co. Ltd., Japan).

### Experiments

The following experiments were conducted in the laboratory at 25 °C under a 15L–9D photoperiod regime. The skeletons of cylinders (150 mm dia., 250 mm high) for holding up the nets were made using wire and covered with one of the six types of colored insect nets described above. For controls, the skeletons were not covered with any type of net. One seedling of Welsh onion was planted in 200-ml plastic cups (100 mm dia., 45 mm high) containing vermiculite. Fourteen-day old potted plants (with two leaves each) were placed in each of the six types of colored net and set in a plastic ventilated cage (304 mm wide, 250 mm dia., 280 mm high) for 24 h. 20 female adults were then released into each ventilated cage. After another 24 h, the number of adults and the feeding marks (abrasion marks) on Welsh onion leaves were counted. The experiment was replicated 10 times. In addition, two each of the five types of 0.8 mm mesh colored insect nets (except black and black type), for a total of 10 test combinations, were selected and placed together 10 cm apart inside a plastic ventilated cage (304 mm wide, 250 mm dia., 280 mm high) with a 10 cm opening. One seedling of Welsh onion was placed inside the insect net, as in the above experiment. In the center of the plastic ventilated cage, 20 female adults were released. The number of adults on Welsh onion leaves was counted after 24 h. This experiment was replicated 6 times.

### Field trials

Two experiments were conducted in a Welsh onion field at the Kyoto Prefectural Agriculture, Forestry, and Fisheries Technology Center in Kameoka City, Kyoto Prefecture from June to August in 2016 (Trial 1) and in 2017 (Trial 2).

### Trial 1

The Welsh onion field was divided into four experimental plots (each 21.6 m^2^, 5.4 m X 4 m). For each of the three test plots, the entire surface of the greenhouse framework (4.0 m width, 5.4 m depth, 2.0 m height) was covered with 0.8 mm mesh red-white, red-red, or white-white types of insect net (Fig. S1). The control plot was not covered with insect net. This experiment was repeated twice. Welsh onion plants were planted on 15 June 2016. The cultivars planted were ‘Shinkujohosonegi’ and ‘Ryokusyu’ with plant and row spacing of 14 cm and 25 cm, respectively. In all test plots, no chemical insecticides were used during the study period.

Plants were monitored once every two weeks, from 5 July to 15 August 2016. We counted the number of *T. tabaci* individuals and leaves damaged by thrips (except on 5 July) on 10 plants in each experimental plot. The numbers of leaves with necrotic spots by IYSV were also counted. Infection of IYSV was confirmed by DAS-ELISA when chlorotic spots were observed on the Welsh onion plants in each treatment. Leaves with chlorotic spots were counted as diseased leaves.

### Trial 2

As in Trial 1, the field was divided into four experimental plots (each 21.6 m^2^, 5.4 by 4 m): plot with only the framework (4.0 m width, 5.4 m depth, 2.0 m height) covered with 0.8 mm mesh red-red insecticidal netting; plot with only the ceiling covered; plot with only the sides covered, and plot not covered with any netting (Fig. S1). This experiment was replicated twice. Welsh onion plants were planted on 13 June 2017. The cultivars planted were ‘Taibyosobutori’. The plant and row spacing was the same as in 2016. Due to the high densities of *T. tabaci* in the untreated area, all experimental plots were sprayed with spinetoram wettable powder on July 20, 2017, and with dinotefuran water-soluble powder on August 15, 2017.

The surveys were conducted as in Trial 1, at approximately two-week intervals during the period of 3 July to 14 August 2017.

All the experiments were performed in accordance with relevant institutional, national, and international guidelines and legislation.

### Analysis

In the single-invasion experiments in the laboratory, the invasion rate through the insect nets was statistically analyzed by Tukey–Kramer multiple comparison test using arcsine transformed values. For the number of feeding marks by thrips, Steel–Dwass multiple comparison test was used. The generalized linear model^50^ was used to analyze the effect of the yarn color on the invasion rate. The invasion rate, r, was set as the dependent variable that is following the binomial distribution. Six types of nets were quantitively systematized based on the amount of red and black fibers that used to make them (SI Appendix, Fig. S2) and used as explanatory variables (Table S1). Logit function, Logit (r) = ln{r/(1–r)}, was used as the link function between invasion frequency and linear expression of explanatory variables. The significance of the regression coefficients was assessed by the Wald test. In the two-nets-choice experiments, the choice rate among the two nets was analyzed by the GLM as well. The choice rate c was set as the dependent variable that is following the binomial distribution. Only the intercept was used as the explanatory variable. The link function and the test of the significance of the intercept were the same as in the single-invasion experiments. In the field trials, thrips populations in each treatment were analyzed for both adults and larvae using the Steel–Dwass multiple comparison test. The rate of leaf damage by thrips and IYSV infection in each treatment were analyzed using the Tukey–Kramer multiple comparison test with arcsine transformed values. Statistical analyses in the multiple comparison were performed using Social Survey Research Information Co., Ltd., and analysis in the GLM was performed using R, which is the language and environment for statistical computing^51^.

### Supplementary Information

Supplementary Information 1.

Supplementary Figures.

Supplementary Tables.

## Data Availability

All raw data and generated data during this study are included in this published article and its supplementary information files.
