# Supplementary material for: Advanced methods for insect nets: red-colored nets contribute to sustainable agriculture
Source: Sci Rep. 2024 Feb 14;14:2255. doi: 10.1038/s41598-024-52108-1 (PMC10866966; doi:10.1038/s41598-024-52108-1)
Supplement: Supplementary file 2 — Supplementary Figures. [file 41598_2024_52108_MOESM2_ESM.pptx]

## Slide 1
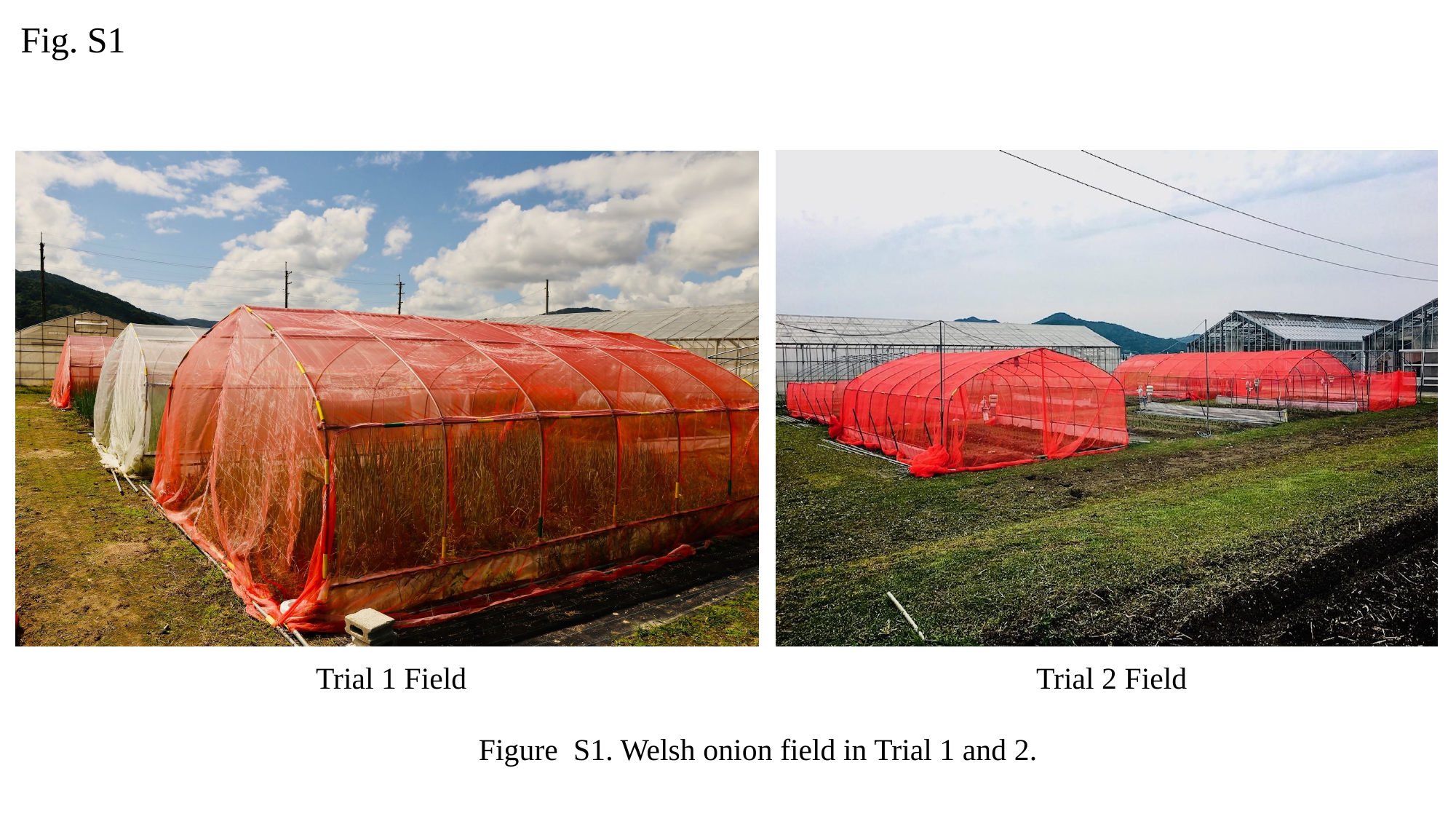

Fig. S1
Trial 1 Field
Trial 2 Field
Figure S1. Welsh onion field in Trial 1 and 2.

## Slide 2
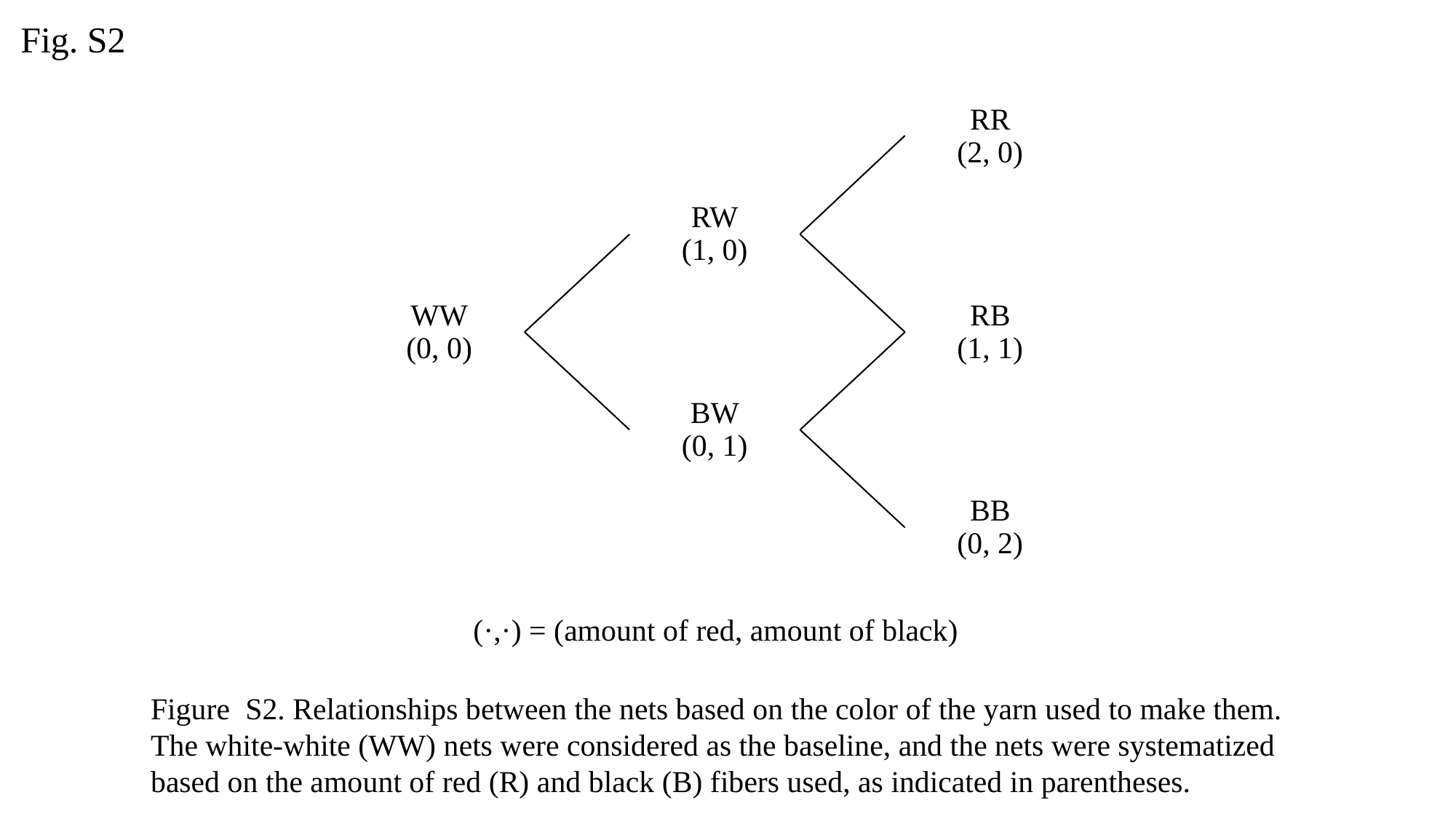

Fig. S2
RR
(2, 0)
RB
(1, 1)
BB
(0, 2)
RW
(1, 0)
BW
(0, 1)
WW
(0, 0)
(·,·) = (amount of red, amount of black)
Figure S2. Relationships between the nets based on the color of the yarn used to make them.
The white-white (WW) nets were considered as the baseline, and the nets were systematized
based on the amount of red (R) and black (B) fibers used, as indicated in parentheses.

## Slide 3
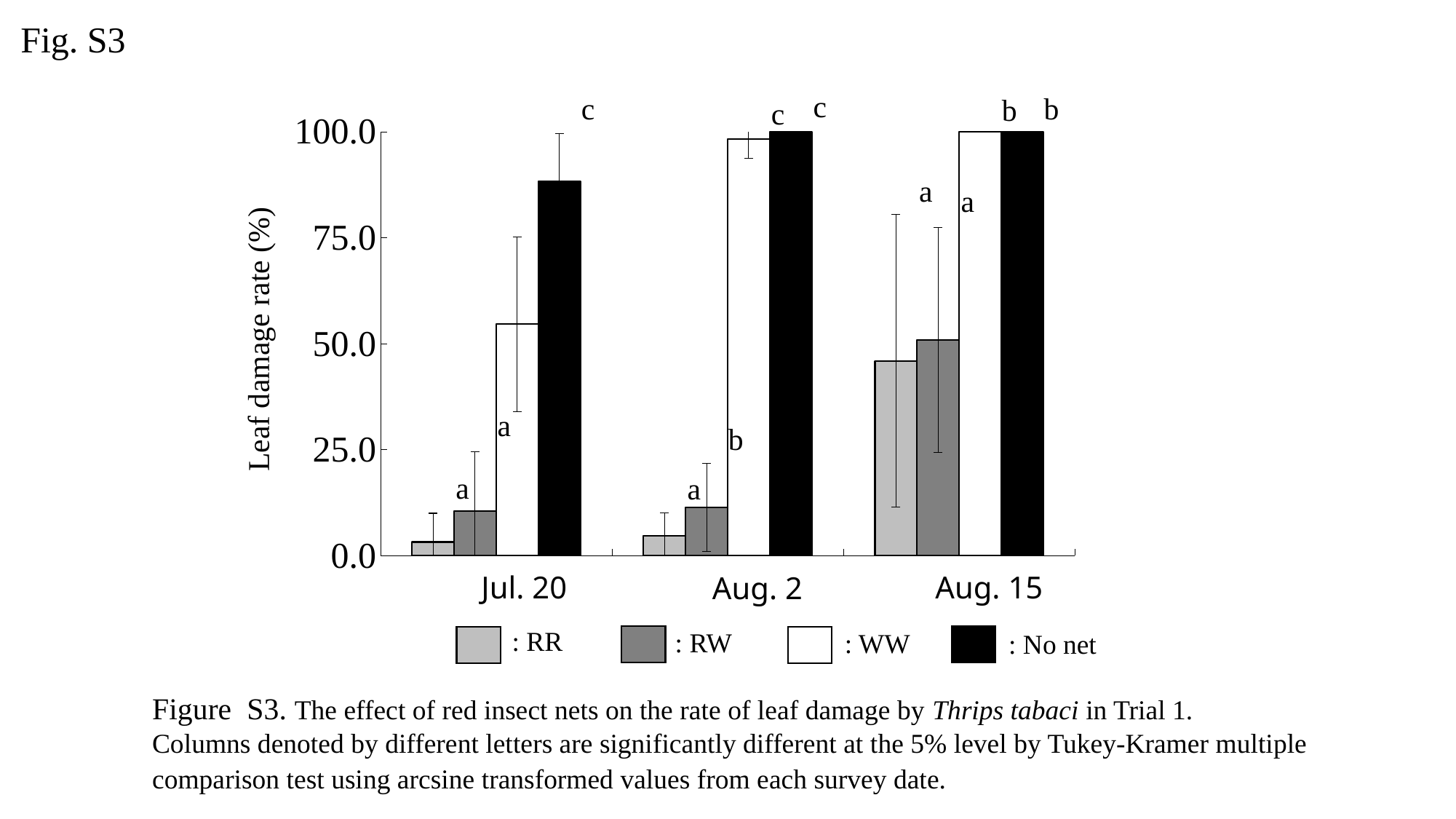

Fig. S3
c
c
b
b
c
### Chart
| Category | 赤赤 | 赤白 | 白 | 無 |
|---|---|---|---|---|
| 7月20日 | 3.189950980392157 | 10.503386762203561 | 54.549963357188425 | 88.33463197878834 |
| ８月２日 | 4.719407634848811 | 11.39494838237098 | 98.23076923076923 | 100.0 |
| ８月15日 | 45.898613791609144 | 50.83049731056697 | 100.0 | 100.0 |a
a
b
Leaf damage rate (%)
a
b
a
a
Jul. 20
Aug. 15
Aug. 2
: RR
: RW
: WW
: No net
Figure S3. The effect of red insect nets on the rate of leaf damage by Thrips tabaci in Trial 1.
Columns denoted by different letters are significantly different at the 5% level by Tukey-Kramer multiple
comparison test using arcsine transformed values from each survey date.

## Slide 4
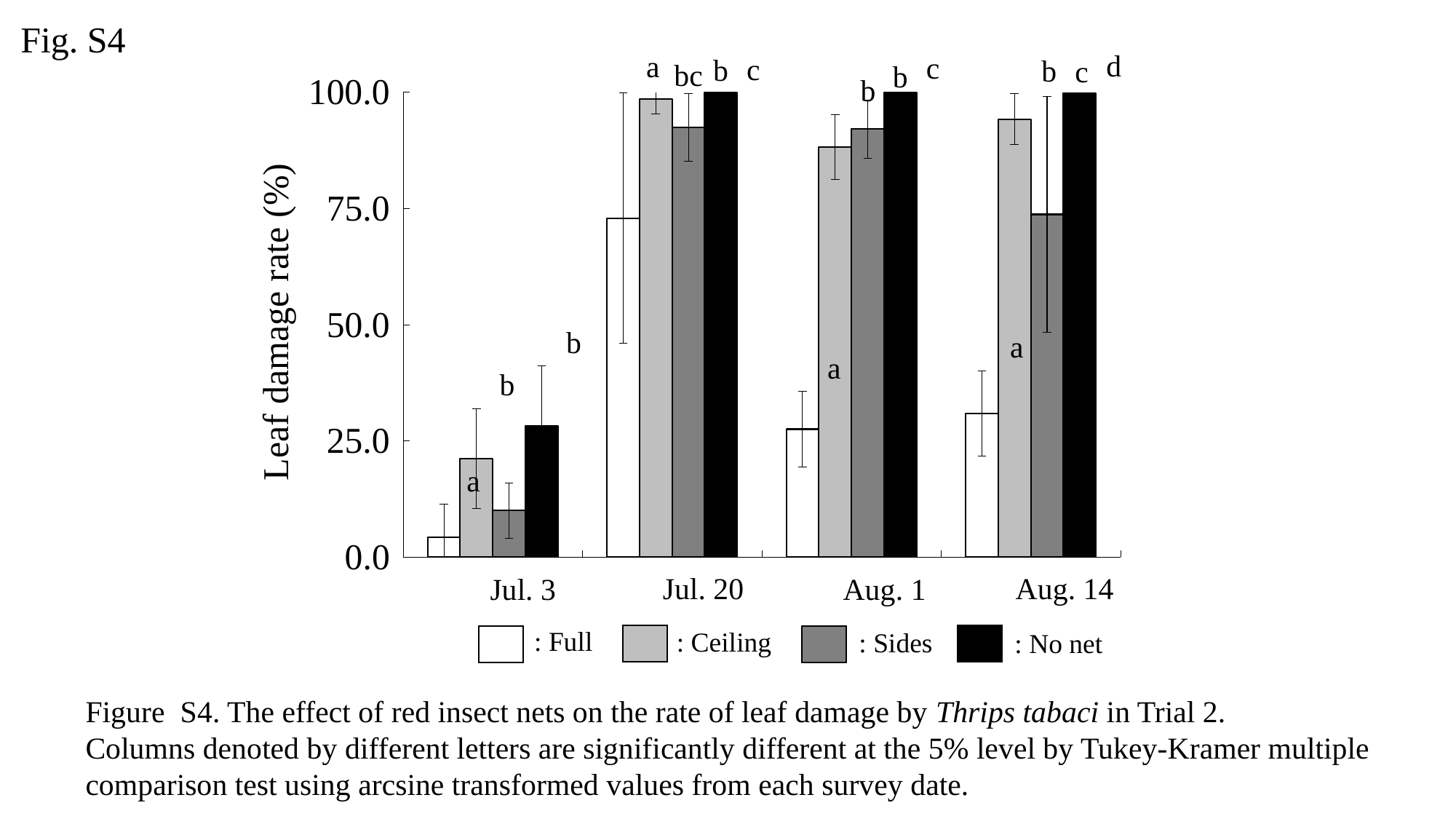

Fig. S4
d
a
c
c
b
b
c
### Chart
| Category | 全面区 | 天井区 | 囲い区 | 無区 |
|---|---|---|---|---|
| 7月３日 | 4.282828282828283 | 21.22924297924298 | 10.037323787323785 | 28.29152791652791 |
| 7月20日 | 72.9139194139194 | 98.46955128205127 | 92.41597291597293 | 100.0 |
| ８月１日 | 27.526009207510754 | 88.20801027747622 | 92.0700721921697 | 100.0 |
| ８月14日 | 30.84395424836601 | 94.1812716652113 | 73.70698051948051 | 99.70588235294117 |bc
b
b
Leaf damage rate (%)
b
a
a
b
c
a
Jul. 20
Aug. 14
Jul. 3
Aug. 1
: Full
: Ceiling
: Sides
: No net
Figure S4. The effect of red insect nets on the rate of leaf damage by Thrips tabaci in Trial 2.
Columns denoted by different letters are significantly different at the 5% level by Tukey-Kramer multiple
comparison test using arcsine transformed values from each survey date.
