# Supplementary material for: Advanced methods for insect nets: red-colored nets contribute to sustainable agriculture
Source: Sci Rep. 2024 Feb 14;14:2255. doi: 10.1038/s41598-024-52108-1 (PMC10866966; doi:10.1038/s41598-024-52108-1)
Supplement: Supplementary file 3 — Supplementary Tables. [file 41598_2024_52108_MOESM3_ESM.docx]

Table S1. Coding of the nets, which is based on the color of the yarn used to make up them (Refer to Fig. S1). These codes were used as explanatory variables in the statistical analysis of the results from single-net-invasion experiments.

|  | Explanatory variable | |
| --- | --- | --- |
| Net | Red yarn | Black yarn |
| RW | 1 | 0 |
| BW | 0 | 1 |
| WW | 0 | 0 |
| RB | 1 | 1 |
| RR | 2 | 0 |
| BB | 0 | 2 |

Table S2. Results of the statistical analysis of the relationships between the color of yarn and the invasion rate in single-net-invasion experiments. Asterisk (*) means that coefficients are significantly different from zero with a significance probability p less than 0.05 (z-test).

| Variables | Coefficients  (vs. White yarn) | SE | p |
| --- | --- | --- | --- |
| (Intercept) | –1.43* | 0.16 | <<0.01 |
| Red yarn | –1.37* | 0.21 | <<0.01 |
| Black yarn | –0.35* | 0.14 | 0.011 |

Table S3. Results of the statistical analysis of data from the choice experiments. In this analysis, the relationships between the net color of the test and control samples were determined for convenient representation. The value of the intercept relates to the probability of choice of the test-color net (for example, the positive value of the intercept means the probability of choice is higher in the test-color net than in the control-color net). Asterisk (*) means that intercept is significantly different from zero with a significance probability p less than 0.05 (z-test).

| Test | Control | Intercept | SE | p |
| --- | --- | --- | --- | --- |
| RW | RB | –0.37 | 0.43 | 0.4 |
| RW | RR | 0.57 | 0.35 | 0.1 |
| RW | BW | –1.70* | 0.38 | <<0.01 |
| RW | WW | –1.42* | 0.39 | <<0.01 |
| RB | RR | –0.44 | 0.43 | 0.3 |
| RB | BW | –1.92* | 0.48 | <<0.01 |
| RB | WW | –1.34* | 0.5 | <<0.01 |
| RR | BW | –1.50* | 0.45 | <<0.01 |
| RR | WW | –2.36* | 0.47 | <<0.01 |
| BW | WW | 1.15* | 0.43 | <<0.01 |

Table S4. Number of insecticide applications in each experimental plot in Trial 2.
